# Gut microbiome regulation in equine animals: current understanding and future perspectives

**DOI:** 10.3389/fmicb.2025.1602258

**Published:** 2025-09-24

**Authors:** Fenglin Li, Xiangyu Kong, Muhammad Zahoor Khan, Lin Wei, Jinjin Wei, Mingxia Zhu, Guiqin Liu, Bingjian Huang, Changfa Wang, Zhenwei Zhang

**Affiliations:** ^1^Liaocheng Research Institute of Donkey High-Efficiency Breeding and Ecological Feeding, College of Agriculture and Biology, Liaocheng University, Liaocheng, China; ^2^State Key Laboratory of Animal Nutrition, College of Animal Science and Technology, China Agricultural University, Beijing, China

**Keywords:** equine, microbial community, metabolism, health, production

## Abstract

The equine intestinal microbiome represents a complex and dynamic ecosystem that fundamentally influences host health and physiological function. This microbial community exhibits distinct compositional and functional variations across different anatomical segments of the intestinal tract, with diversity and abundance patterns shaped by host genetics, dietary inputs, and environmental conditions. The resident microbiota performs essential functions in feed fermentation, nutrient metabolism, pathogen exclusion, and immunological programming. This review synthesizes current knowledge regarding the core taxonomic and functional attributes of the equine intestinal microbiome, examining interspecies variation and conservation patterns. We evaluate key determinants of microbial community assembly and regulation, while examining mechanistic links between microbiota composition and host health outcomes. Through critical analysis of existing literature, this work provides an integrated framework for understanding the equine gut microbiome, with implications for clinical intervention strategies and evidence-based approaches to promote intestinal health in equine.

## 1 Introduction

Unlike ruminants, equines use cecal fermentation—a microbial system similar to the rumen that aids digestion, strengthening the intestinal protective barrier and immune (Wunderlich et al., 2023; Zhang et al., 2022a; Kou et al., 2024a; Dicks et al., 2014). The equine gastrointestinal tract represents a complex microecological environment populated by diverse communities of anaerobic bacteria, fungi, parasites, and viruses that collectively enable efficient degradation of low-quality fibrous substrates, thereby providing substantial energy to the host (Raspa et al., 2024; Wunderlich et al., 2023; Liu et al., 2019). The intestinal microbiota serves critical functions beyond nutrient processing, acting as a fundamental determinant of host health through disease prevention and immunomodulatory mechanisms (Sävilammi et al., 2024; Ma et al., 2023; Mach et al., 2022). Initial microbial colonization commences perinatally through vertical transmission via the birth canal, with subsequent community development influenced by maternal contact and environmental exposure (Zhang et al., 2023; Barko et al., 2018).

Consistent with patterns observed across mammalian taxa, the establishment of a diverse and stable gut microbiota is essential for optimal digestive efficiency, nutrient bioavailability, immune system maturation, and metabolic homeostasis throughout equine development (Xing et al., 2025; Matsuki et al., 2016; Mueller et al., 2015). Contemporary research has elucidated distinct microbial signatures across anatomical compartments of the equine digestive tract (Lan et al., 2025; Wang et al., 2025a, 2025b; Reed et al., 2021). The gastrointestinal system is functionally divided into upper and lower regions, with the foregut (stomach and small intestine) exhibiting greater microbial variability due to the substantial influx of environmental bacteria through dietary intake (Kauter et al., 2019). Microbial community composition demonstrates significant responsiveness to environmental factors and host physiological status (Khan et al., 2025), reflecting the dynamic and symbiotic nature of host–microbe interactions that maintain intestinal homeostasis and systemic health (Ang et al., 2022).

Host-microbe crosstalk is mediated primarily through microbial metabolite production. Intestinal microorganisms catabolize dietary substrates and endogenous compounds to generate bioactive metabolites, including short-chain fatty acids (SCFAs) and phytoestrogens. In equines, microbial fermentation in the cecum and colon contributes approximately 30–40% of total energy requirements through SCFA production (Garber et al., 2020), underscoring the metabolic significance of these compounds for cardiovascular and systemic health (Mach et al., 2022). Additionally, the microbiota orchestrates immune homeostasis by preserving mucosal barrier integrity, promoting immune cell differentiation and activation, and maintaining colonization resistance against pathogenic microorganisms through competitive exclusion and antimicrobial compound synthesis, thereby sustaining microbial equilibrium and preventing disease manifestation.

The intestinal microbiota plays a pivotal role in equine health; however, its composition and functional capacity are significantly modulated by multiple intrinsic and extrinsic factors, including breed characteristics, reproductive status (pregnancy and lactation stage), dietary composition, age, anatomical location within the gastrointestinal tract, and environmental conditions (Qin et al., 2025; Su et al., 2024; Xing et al., 2025; Sha et al., 2024; Zhou et al., 2023a, 2023b; Li et al., 2022a; Zhao et al., 2020). The advent of high-throughput sequencing technologies has revolutionized equine gut microbiome research, enabling increasingly sophisticated analytical approaches. Current methodologies primarily employ 16S rRNA gene amplicon sequencing and shotgun metagenomics, with universal primers targeting conserved regions of the rRNA gene followed by sequencing of internal transcribed spacer regions, techniques extensively validated in fungal taxonomic studies (Zhang et al., 2022b). Recent advances in computational binning algorithms have facilitated the reconstruction of metagenome-assembled genomes (MAGs) from metagenomic datasets, substantially expanding the reference genome repository for mammalian intestinal microorganisms (Kou et al., 2024b). Despite these technological advances, significant knowledge gaps remain regarding the core microbiota of equine species and their functional significance in host physiology.

This review synthesizes current understanding of the fundamental intestinal microorganisms in equine species, elucidates their functional roles, and examines the impact of various intervention strategies on microbial community structure and function. The comprehensive analysis presented herein provides a foundation for developing targeted approaches to optimize microbial-mediated feed utilization efficiency and enhance disease resistance in equine populations.

## 2 Literature search methodology

A comprehensive literature review was conducted to examine gut microbiome regulation in equine animals using multiple electronic databases, including Google Scholar, PubMed, XMOL, and Web of Science, to ensure comprehensive coverage of relevant publications. The following keywords and search terms were employed in various combinations: “equine gut microbes,” “horses,” “donkeys,” “ruminants,” “microbiota effect on equine health and production,” “gut microbiota composition,” and “factors affecting gut microbiota.” Only peer-reviewed articles published in English-language SCI journals were included in this review, focusing specifically on studies examining equine gut microbiome and related health outcomes. Publications in languages other than English, book chapters, conference papers, theses, dissertations, and unpublished data were systematically excluded from the analysis. This systematic approach ensured that only high-quality, peer-reviewed research was included in the review, providing a robust foundation for analysis and synthesis of current knowledge regarding equine gut microbiome regulation.

## 3 Core microbiota of equine animals

**Figure 1:**
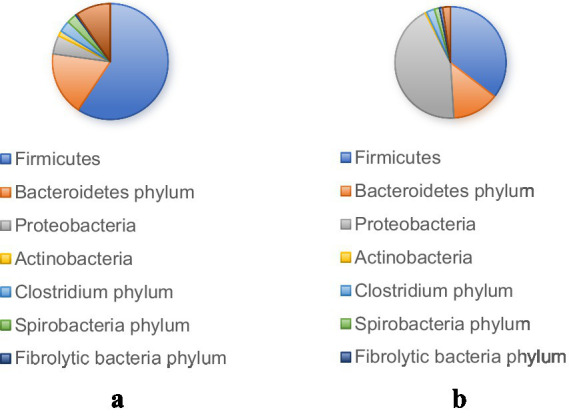
Microbial phyla with a relative abundance (>1%) in donkey **(a)** and horse **(b)** digestive tract.

The core microbiota of the equine intestinal tract comprises microbial species that dominate the gut microbial community and significantly influence the health and physiology of equine animals. These microorganisms are characterized by their functional versatility and adaptability, enabling their persistent presence and vital role in the host’s digestive system. They contribute to nutrient metabolism, immune modulation, and the maintenance of intestinal homeostasis. However, the specific composition of the core microbiota is not uniform and may vary due to factors such as individual genetic differences, environmental conditions, and dietary practices (Costa and Weese, 2012). Understanding these variations is essential for developing tailored nutritional and management strategies to optimize the health and performance of equine animals. For ease of reference, the comparative abundance of gut microbiota in donkeys and horses has been presented in Table 1 and Figure 1, which are sourced from previously published articles (Liu, 2020; Su et al., 2020; Zhang et al., 2020; Liu et al., 2019).

**Table 1:** Comparison of relative richness (>1%) in the intestinal microbiota of horses and donkeys.

Intestinal area	Jejunum		Ileum		Cecum		Dorsal colon		Abdominal colon		Rectum (feces)	
	Donkey	Horse	Donkey	Horse	Donkey	Horse	Donkey	Horse	Donkey	Horse	Donkey	Horse
Firmicutes	50.3% ~ 85.6%	67.3% ~ 79.3%	60.3% ~ 81.8%	73.5% ~ 78.8%	38.3% ~ 45.0%	38.6% ~ 40.5%	43.2% ~ 56.8%	45.3% ~ 47.6%	45.6% ~ 62.6%	48.1% ~ 54.2%	54.5% ~ 56.1%	50.3% ~ 69.0%
Bacteroidetes phylum	1.0% ~ 9.4%	0.7% ~ 3.7%	<1%	0.7% ~ 1.8%	41.7% ~ 48.6%	45.7% ~ 47.5%	33.6% ~ 42.0%	42.7% ~ 43.4%	31.1% ~ 37.4%	33.5% ~ 36.9%	33.4% ~ 34.2%	5.9% ~ 15.29%
Proteobacteria	11.5% ~ 12.4%	14.1% ~ 15.6%	9.4% ~ 16.2%	17.3% ~ 17.5%	3.1% ~ 4.6%	0.5% ~ 1.7%	3.3% ~ 4.6%	0.3% ~ 1.1%	0.9% ~ 1.7%	0.4% ~ 1.6%	3.4% ~ 4.2%	1.1% ~ 2.82%
Actinobacteria	0.8% ~ 1.7%	0.4% ~ 1.1%	1.1% ~ 1.5%	<1%	<1%	<1%	<1%	<1%	<1%	<1%	<1%	<1%
Clostridium phylum	<1%	0.6% ~ 1.1%	<1%	<1%	<1%	<1%	<1%	<1%	<1%	<1%	5.6% ~ 11%	1.3% ~ 4.53%
Spirobacteria phylum	<1%	<1%	<1%	<1%	3.90% ~ 5.30%	0.3% ~ 3.6%	1.3% ~ 1.5%	1.2% ~ 3.4%	1% ~ 1.8%	0.7% ~ 1.9%	5.32% ~ 5.7%	0.5% ~ 1.24%
Fibrolytic bacteria phylum	<1%	<1%	<1%	<1%	<1%	0.2% ~ 2.5%	0.8% ~ 1.3%	0.4% ~ 1.7%	<1%	0.2% ~ 1.4%	<1%	<1%
Verrucomycota phylum	<1%	<1%	<1%	<1%	<1%	0.8% ~ 3.8%	<1%	0.7% ~ 2.4%	<1%	1.2% ~ 6.1%	2.1% ~ 3.55%	0.4% ~ 3.28%

The intestinal tracts of horses and donkeys are predominantly colonized by two major bacterial phyla: *Firmicutes* and *Bacteroidetes*, which collectively constitute the majority of the microbial community (Zhang et al., 2022c). Notably, Liu (2020) demonstrated significant interspecies variations in the relative abundance of these dominant phyla, reporting that *Firmicutes* and *Bacteroidetes* account for 87.91 and 78.50% of the total microbial population in donkeys and horses, respectively. Furthermore, the ratio of *Firmicutes* to *Bacteroidetes* differs markedly between these species. In donkeys, this ratio approximates 1:1, whereas in horses, it is approximately 0.8:1. These proportional differences are of particular significance, as they are believed to influence the host’s capacity for energy extraction from dietary sources and may consequently be associated with metabolic conditions such as obesity. Specifically, an elevated proportion of *Firmicutes* relative to *Bacteroidetes* has been correlated with enhanced energy harvesting capabilities from the diet.

The functional specialization of these bacterial phyla underlies their importance in equine digestive physiology. *Bacteroidetes* serves as a key player in the metabolism of complex compounds, including steroids, bile acids, and polysaccharides, and is particularly crucial for cellulose degradation in herbivorous animals (Costa and Weese, 2012). In contrast, *Firmicutes* primarily functions in the hydrolysis of carbohydrates and proteins (Spence et al., 2006), establishing itself as the predominant bacterial group responsible for carbohydrate utilization in herbivorous species (Xu et al., 2003). Based on these distinct functional characteristics, it has been hypothesized that horses may possess superior metabolic capabilities for processing roughage, while donkeys may demonstrate greater efficiency in metabolizing concentrate feeds. This functional divergence reflects the evolutionary adaptations of these species to their respective dietary niches and feeding behaviors.

The relationship between microbial composition and gastrointestinal health has been well-documented in equine research. Costa et al. (2012), utilizing 16S rDNA sequencing technology, revealed that healthy horses maintain significantly higher concentrations of *Firmicutes* in their intestinal microbiota compared to horses suffering from colitis. This finding suggests a strong association between microbial community structure and gut health status in equine species. Moreover, metagenomic analysis conducted by Guo et al. (2023) identified *Clostridium* and *Bacteroidetes* as the most abundant genera in specific populations. *Clostridium*, classified as cellulose-degrading bacteria, plays a multifaceted role in the intestinal ecosystem. These bacteria not only decompose cellulose to provide fermentation substrates for acid-producing bacteria but also utilize succinate produced by other microorganisms to generate acetate and propionate. Through these metabolic processes, *Clostridium* contributes to gut microbiota stabilization and intestinal pH regulation, thereby creating favorable conditions for microbial growth and host health.

Interspecies and intraspecies variations in gut microbiota composition have been consistently observed across different equine populations. Bulmer et al. (2019) conducted a comprehensive study involving 20 horse populations and found that *Firmicutes* dominated the microbial community in 18 of these populations. In contrast, *Bacteroidetes* and *Proteobacteria* were less prevalent, with *Proteobacteria* representing less than 1% of the total relative abundance. Within the *Firmicutes* phylum, the class *Clostridia* and the order *Bacillales* were predominantly represented, with additional contributions from *Lactobacillus* species. Similarly, comparative studies between Mongolian horses and purebred horses have revealed that while overall microbial diversity, richness, and uniformity remain relatively consistent, significant differences exist in the specific bacterial species composition (Brulc et al., 2009). These variations likely reflect the unique physiological adaptations that different horse breeds have developed in response to their respective environmental conditions, ultimately influencing the structure and function of their gut microbiota.

Research focusing specifically on donkey populations has provided valuable insights into species-specific microbial patterns. Zhang et al. (2024) investigated the intestinal microbiota of three distinct donkey populations: Yunnan, Dezhou, and Qinghai donkeys. Their findings confirmed that *Firmicutes* and *Bacteroidetes* remain the dominant bacterial groups across all three populations, with *Rikenellaceae* emerging as a notable genus within the *Bacteroidetes* phylum. The predominance of *Firmicutes* in donkey populations may be attributed to the unique cellular structure of these bacteria, which enables them to withstand the harsh intestinal environment while efficiently metabolizing complex carbohydrates such as cellulose and pectin. This metabolic capability is essential for the production of short-chain fatty acids (SCFAs), which are vital for host energy supply and maintenance of intestinal health (Guo et al., 2022).

The comprehensive understanding of intestinal microbiota composition and functional roles in equine species is paramount for advancing scientific breeding practices and developing effective strategies for intestinal disease prevention. The documented differences between horses and donkeys, as well as variations among different breeds, underscore the critical need for species-specific and breed-specific microbiome management approaches to optimize feed utilization efficiency and promote overall gut health. Consequently, continued research efforts are essential to deepen our understanding of these complex microbial communities and their broader implications for equine health, disease prevention, and performance optimization. Future investigations should focus on elucidating the mechanistic relationships between microbiota composition, dietary interventions, and health outcomes to develop evidence-based management strategies for equine populations.

## 4 Functions of intestinal microbes in equine animals

The hindgut of horses is a complex anaerobic fermentation system that inhabits a large microbial community and is crucial for maintaining health and digestive efficiency. These microorganisms promote nutrient absorption and energy supply by fermenting plant cellulose (Santos et al., 2013) and producing volatile fatty acids (VFAs, such as acetic acid, propionic acid, and butyric acid) (He et al., 2020). The weakly acidic environment of the cecum (pH 6–7) is conducive to microbial activity, and its mucosal epithelium forms a symbiotic barrier with the microbiota, inhibiting pathogens and enhancing immune defense.

In addition, gut microbiota may affect the athletic performance of horses. Research has found that the abundance of bacteria such as *Helicobacter* and *Ruminococcus* in horse racing is relatively high, and the increase in short chain fatty acid (SCFA) production may provide additional energy (Li et al., 2023). However, the widespread use of antibiotics may lead to the accumulation of resistance genes (Pimenta et al., 2023), which requires careful management to maintain microbial community function.

In summary, the gut microbiota of horses has multiple important functions, including digesting nutrients from feed, maintaining gut health, promoting immune protection, and potentially affecting bodily functions. Further research is needed to deepen our understanding of these interactions and their implications for horse health and management.

### 4.1 Role of gut microbiota in digestion and degradation of nutrients

The gut microbiota of equine species has co-evolved with their hosts and is intricately influenced by their diet. These microorganisms, which evolved alongside the ancestors of horses, have adapted to the challenges posed by the equine digestive system and play a critical role in the digestion and utilization of nutrients. For instance, lipid digestion in the intestinal tract of Equus species is facilitated by microbial communities that not only support the metabolism of fats but also promote fat deposition. Research has demonstrated that the colonization of *Firmicutes* species in the gut is associated with obesity in equines, while the presence of other microbial groups, such as *Bacteroides*, has been linked to reduced fat accumulation. *Firmicutes* are primarily involved in the fermentation of polysaccharides and cellulose, producing SCFAs and other caloric compounds, which contribute to the energy balance and fat storage (Bäckhed et al., 2004; Ley et al., 2005). Consequently, a higher population of *Firmicutes* correlates positively with the degree of obesity in horses. In contrast, *Bacteroidetes* are involved in the breakdown of starch and fiber, yielding energy while simultaneously reducing fat deposition, thus inversely correlating with obesity levels (Araújo et al., 2020). These findings highlight the potential for microbial manipulation to regulate body weight and improve nutritional strategies in equine production (Schwiertz et al., 2010). However, it should be pointed out that the proportion of *Firmicutes/Bacteroidetes* (F/B) in human and animal microbiome research is controversial and requires specific analysis in practical applications.

#### 4.1.1 Role of gut microbiota in lipid metabolism

Gut microbiota plays a crucial role in regulating lipid metabolism through the production of various metabolites. Key microbial species such as *Bifidobacterium*, *Lactobacillus*, *Prevotella*, and *Streptococcus*, as well as *Bacteroides* and *Escherichia coli*, are known to produce SCFAs, which are pivotal in modulating lipid metabolism (Detman et al., 2019; Jian et al., 2022). SCFAs, including acetic acid, propionate, and butyric acid, significantly influence fat metabolism in animals (Li et al., 2022b). These metabolites are absorbed by intestinal cells, where acetate is converted into adenosine monophosphate (AMP) and acetyl-CoA, thereby regulating the AMPK/PGC-1α/PPARα signaling pathway (Jian et al., 2022).

**Figure 2:**
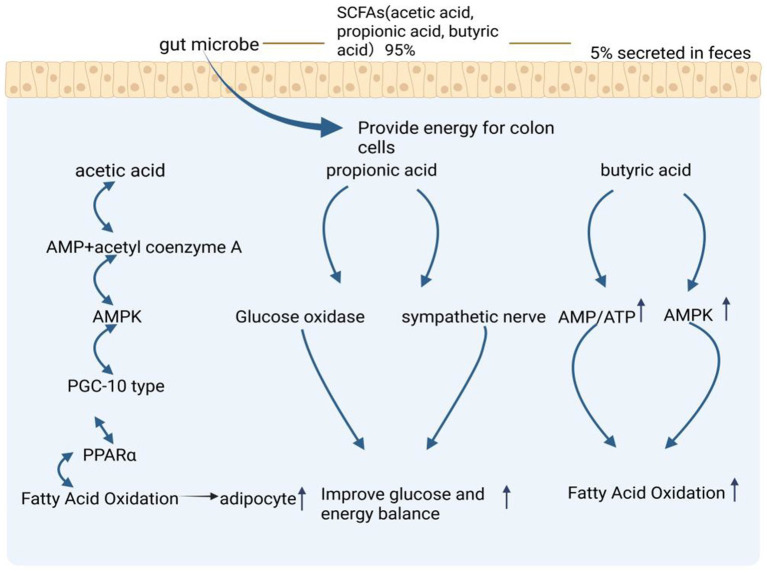
Regulation of lipid metabolism mechanism by short chain volatile acids (SCFAs) produced by gut microbiota metabolism. The metabolic pathways of SCFAs- specifically acetic acid, propionic acid, and butyric acid - which are produced by gut microbes, with 95% utilized by colon cells and 5% secreted in feces. The figure shows three main pathways: the acetic acid pathway leading to fatty acid oxidation through AMPK and PPARα, the propionic acid pathway improving glucose and energy balance via glucose oxidase and sympathetic nerve activation, and the butyric acid pathway enhancing fatty acid oxidation through AMP/ATP and AMPK signaling. The figure is created with BioRender.com.

Among the SCFAs, propionate and butyrate play critical roles in maintaining glucose and energy homeostasis by promoting intestinal gluconeogenesis and enhancing sympathetic nervous activity. Additionally, acetate and butyrate have been shown to directly increase the phosphorylation and activity of hepatic AMPK by modulating the AMP/ATP ratio, which in turn upregulates PPARα target genes. This results in enhanced fatty acid oxidation and improved glycogen storage (Yin et al., 2016). Butyrate, in particular, functions as a HDAC inhibitor, thereby influencing lipolysis and promoting fat oxidation. It also plays a significant role in modulating the physiological functions of adipose tissue and other organs, with a noted inhibitory effect on fat deposition (Figure 2; Yin et al., 2016).

Acetic acid, produced by gut microbiota, has been identified as a precursor for the synthesis of long-chain fatty acids such as palmitic acid and stearic acid, which further regulate hepatic fatty acid metabolism (Araújo et al., 2020). Furthermore, *E. coli* strain RB 01 has been shown to produce acetic acid that reduces intracellular triglyceride and cholesterol ester accumulation, as well as decreases lipid droplet size. This mechanism impedes bacterial ability to increase intestinal lipid consumption, thereby reducing lipid accumulation and secretion, and ultimately leading to enhanced lipid utilization within the intestinal tract (Broeders et al., 2015).

#### 4.1.2 Role of gut microbiota in the metabolism of bile acids

**Figure 3:**
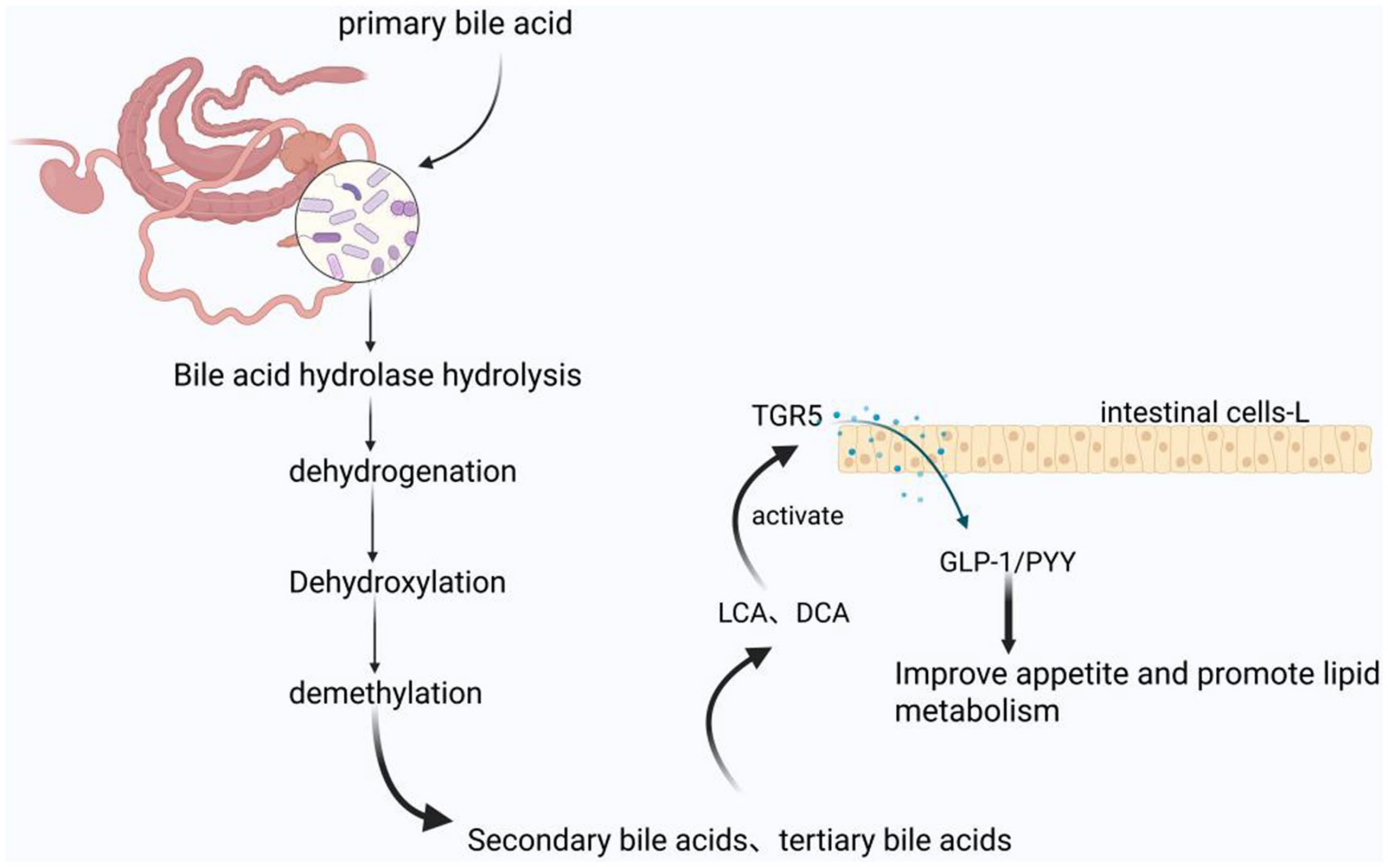
Bile acid regulated lipid metabolism mechanism produced by gut microbiota metabolism in equine animals: The products produced by microbial hydrolysis, hydrogenation, dehydroxylation, and demethylation of primary bile acids are activators of TGR5, thereby improving animal appetite and lipid metabolism. The figure is created with BioRender.com.

The gut microbiota plays a pivotal role in bile acid metabolism through complex biotransformation processes. Primary bile acids, synthesized hepatically from cholesterol, undergo extensive microbial modification within the intestinal tract. Intestinal bacteria facilitate the biotransformation of these primary bile acids through enzymatic processes including deconjugation (hydrolysis), 7α-dehydroxylation, dehydrogenation, and oxidation, resulting in the formation of secondary bile acids such as lithocholic acid (LCA) and deoxycholic acid (DCA). These secondary bile acids function as endogenous ligands for the G-protein coupled bile acid receptor TGR5 (GPBAR1) (Virtue et al., 2019). Upon TGR5 activation, a downstream signaling cascade is initiated that stimulates the secretion of incretin hormones, specifically glucagon-like peptide-1 (GLP-1) and peptide YY (PYY), from intestinal enteroendocrine L cells. This hormonal response subsequently modulates animal appetite and lipid metabolism (Figure 3; Chen et al., 2011). Beyond bile acid biotransformation, the gut microbiota exerts metabolic influence through additional substrate-specific pathways that converge on lipid homeostasis regulation. Complementing the bile acid-TGR5-incretin axis, microbial amino acid metabolism represents another critical mechanism by which the intestinal microbiome modulates host metabolic physiology (Figure 3).

In addition, studies in mice have demonstrated that approximately 4 to 6% of tryptophan is directly metabolized by gut microbiota into various derivatives, including indole compounds (Neavin et al., 2018). These metabolites affect the expression of small RNA molecules such as miR-181 in adipocytes, and overexpression of miR-181 has been shown to restore adipocyte differentiation and lipid accumulation, indicating that the inhibition of miR-181 mediates some anti adipogenic effects of diet induced obesity (DOS) in cultured white adipocytes (Virtue et al., 2019). These findings indicate that miR-181 plays an important role in fat deposition, and gut microbiota can affect the expression of these small molecules by metabolizing tryptophan. However, further research is needed to determine whether tryptophan and its derivatives regulate fat metabolism through the AhR and miR-181 pathways in horse species.

#### 4.1.3 Role of gut microbiota in nitrogen metabolism

**Figure 4:**
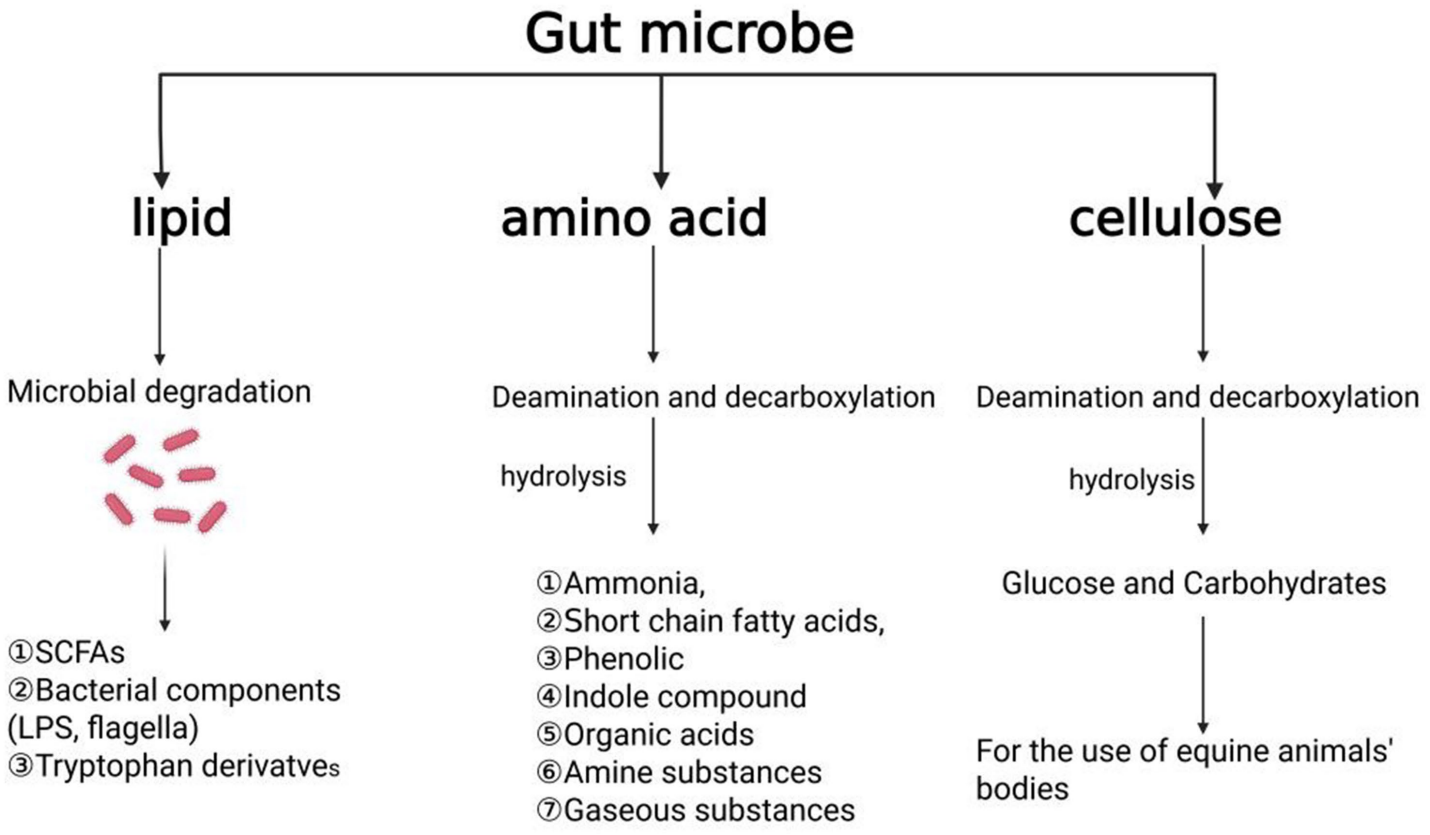
Summary of digestion and degradation of three major nutrients in feed by equine animals, including lipid, amino acid and cellulose. The figure is created with BioRender.com.

Protein is an essential nutrient for the growth and development of equine species. Nitrogen-containing substances are critical for bacterial protein synthesis. The gut microbiota plays a pivotal role in nitrogen metabolism, particularly through the degradation of amino acids. Most intestinal microorganisms are capable of directly utilizing the free amino acids available in the intestine. Microorganisms in the digestive tract employ various mechanisms to adapt to challenging conditions such as high pH, nutrient scarcity, and digestive enzyme activity. They contribute to the synthesis of essential nutrients, which may be utilized by the host, and facilitate the deamination and decarboxylation of amino acids, resulting in the production of ammonia, short-chain fatty acids, organic acids, phenolic compounds, and gases (Zhang et al., 2025; Chaucheyras-Durand et al., 2022; Lin et al., 2017; Blachier et al., 2007; Radecki and Yokoyama, 2001). While the primary sources of amino acids in monogastric animals are the diet and endogenous proteins, the small intestine in equines is a key site for nutrient metabolism, but its efficiency in utilizing amino acids is limited (Metges, 2000; Torrallardona et al., 2003). In some instances, microbial protein synthesis in the foregut may lead to the excretion of amino acids, which could represent a loss of potential nutrition for the host. However, it remains unclear whether the synthesis of microbial proteins competes with the nutritional demands of the intestinal mucosa, warranting further investigation. Some studies also suggest that the amino acid biosynthesis by intestinal microbes in monogastric animals may help regulate the body’s amino acid balance (Metges, 2000; Fuller and Reeds, 1998; Metges and Petzke, 2005), however, the exact role of these newly synthesized microbial amino acids in regulating body amino acid metabolism remains uncertain (Costa et al., 2012). The summary of digestion and degradation of lipid, amino acid and cellulose in feed by equine animals is presented in Figure 4.

### 4.2 Role of gut microbiota in disease prevention and health maintenance

The gut microbiota plays a central role in maintaining the stability of the microbiota in the health of equine animals, while an imbalance in the microbiota directly leads to various intestinal diseases. The gut of healthy horses is dominated by *Firmicutes* (68%), but in horses with colitis, the abundance of *Bacteroidetes* abnormally increases to 40%, while *Firmicutes* decreases to 30%. The fundamental transformation of the horizontal structure of this phylum leads to a decrease in cellulose degradation ability and an increase in the release of inflammatory factors, directly damaging the colon mucosa, confirming that dysbiosis (rather than a single pathogen) is the core cause of colitis (Costa et al., 2012). The development of diarrhea in foals is directly attributed to the collapse of microbial diversity: the abundance of *Clostridium* and *Lactobacillaceae* in diarrheal foals significantly decreases, weakening the intestinal barrier, while the abundance of *actinomycetes* and *Micrococcus*, which have immune regulatory functions, further weakens the pathogen’s resistance, ultimately leading to excessive proliferation of opportunistic pathogens and causing watery diarrhea (Schoster et al., 2017; De la Torre et al., 2019). In the pathogenesis of colic, the ratio of *Firmicutes* to *Proteobacteria* (≥0.95) directly reduces the risk of onset by producing anti-inflammatory short chain fatty acids and stabilizing intestinal peristalsis, while an imbalanced ratio leads to intestinal motility disorders and mucosal inflammation (Stewart et al., 2019); It is worth noting that deworming drugs (such as fenbendazole) further disrupt the buffering capacity of the microbiota by inhibiting the abundance of *Bacteroidetes*, indirectly increasing susceptibility to colic (Hillyer et al., 2002; Walshe et al., 2019). These studies collectively reveal that diseases such as colitis, diarrhea, and colic all begin with dysbiosis of the microbiota structure (inversion of phylum proportions or absence of taxonomic groups), and a decrease in microbial diversity directly weakens the intestinal immune metabolism homeostasis. In the future, it is necessary to focus on analyzing the colonization mechanisms of specific bacterial communities (such as *lactobacilli*), and restore ecological balance through microbial transplantation or targeted probiotic intervention, providing new strategies for disease prevention and control.

### 4.3 Gut microbiota role in immune response

The intestinal barrier in horses is a dynamic mucosal layer composed of various components that interact with multiple stimuli to elicit an immune response. This barrier covers the entire intestinal surface, serving as the first line of defense, and simultaneously functions as a complex ecosystem consisting of microorganisms, such as bacteria, fungi, and viruses, along with mucus and nutrients (Quigley, 2017). These microorganisms meet their nutritional needs by fermenting indigestible carbohydrates, including oligofructose, inulin, and galactose (Nishida et al., 2018). Intestinal microbiota regulates hormone release within the intestinal tract, contributing to pathogen resistance, promoting vitamin synthesis and absorption, and modulating neural signals that impact metabolism, physiological health, and immune function (Mangiola et al., 2018).

The interaction between gut microbiota and the host immune system occurs through various pathways. Notably, microbial metabolism of SCFAs, such as propionic acid and butyric acid, plays a central role. These SCFAs interact with G protein-coupled receptors (GPCRs), inhibiting histone deacetylase (HDAC) activity and regulating intestinal immune functions. Propionic acid and butyric acid activate GPR43 and GPR109A receptors on the surface of intestinal epithelial cells, respectively. Upon activation, these receptors trigger pro-inflammatory responses and initiate signaling pathways involving interleukin-18 (IL-18), which helps maintain epithelial integrity. Additionally, activation of GPR109A induces the secretion of IL-10, promoting the differentiation of regulatory T cells (Singh et al., 2014; Balakrishnan et al., 2021). SCFAs, as potent HDAC inhibitors, also regulate the expression of transforming growth factor beta (TGF-*β*) in intestinal epithelial cells (IECs), influencing T-cell differentiation into effector T cells and Tregs (Yang et al., 2020), as well as enhancing mTOR-S6K activity crucial for T-cell differentiation (Park et al., 2015). Thus, SCFAs are vital for maintaining intestinal barrier function by promoting IEC proliferation, differentiation, and reducing apoptosis.

**Figure 5:**
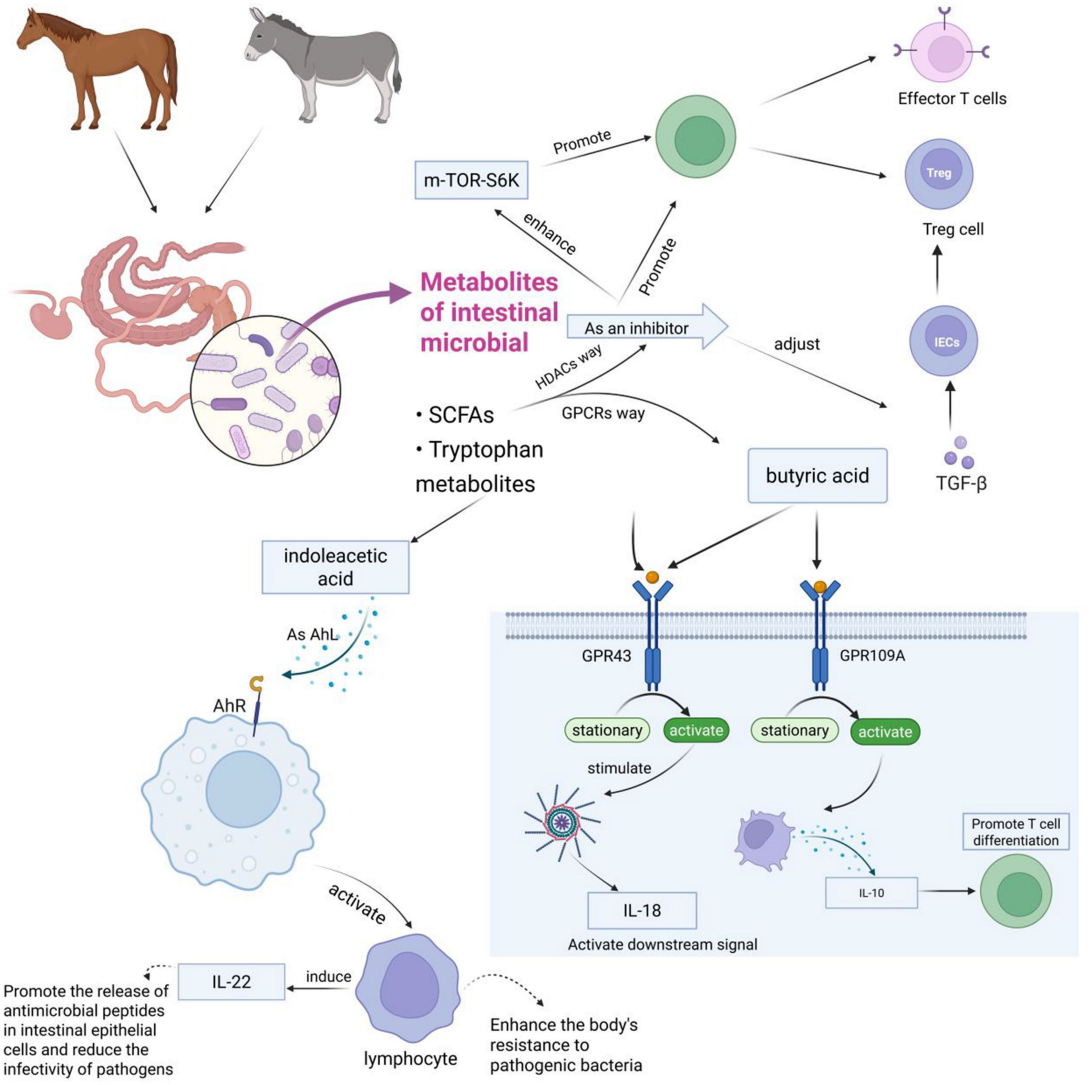
This figure illustrates how intestinal microbial metabolites (primarily SCFAs and tryptophan metabolites) from horses and donkeys influence immune responses through various signaling pathways. The diagram shows several key pathways: the mTOR-S6K pathway affecting T cells (Effector T cells and Treg cells), the butyric acid pathway working through GPR43 and GPR109A receptors to produce IL-1β and IL-10 respectively, and the indoleacetic acid pathway activating AHR to stimulate lymphocytes and IL-22 production, ultimately enhancing antimicrobial defense and T cell differentiation. The figure is created with BioRender.com.

Tryptophan metabolites also play an essential role in immune regulation, enhancing immune function and cell proliferation through activation of the aryl hydrocarbon receptor (AhR) (Ngui et al., 2020). AhR, a sensor for microbial metabolites, protects the host against chemicals and pathogens (Roager and Licht, 2018). In the gut, AhR activation mediates the development of innate and intraepithelial lymphocytes, exerting antibacterial and anti-inflammatory effects (Gutiérrez-Vázquez and Quintana, 2018). Many microbial-derived tryptophan metabolites have been shown to activate AhR, further underscoring the importance of microbial metabolites in host–microbe interactions within the immune system (Figure 5).

**Figure 6:**
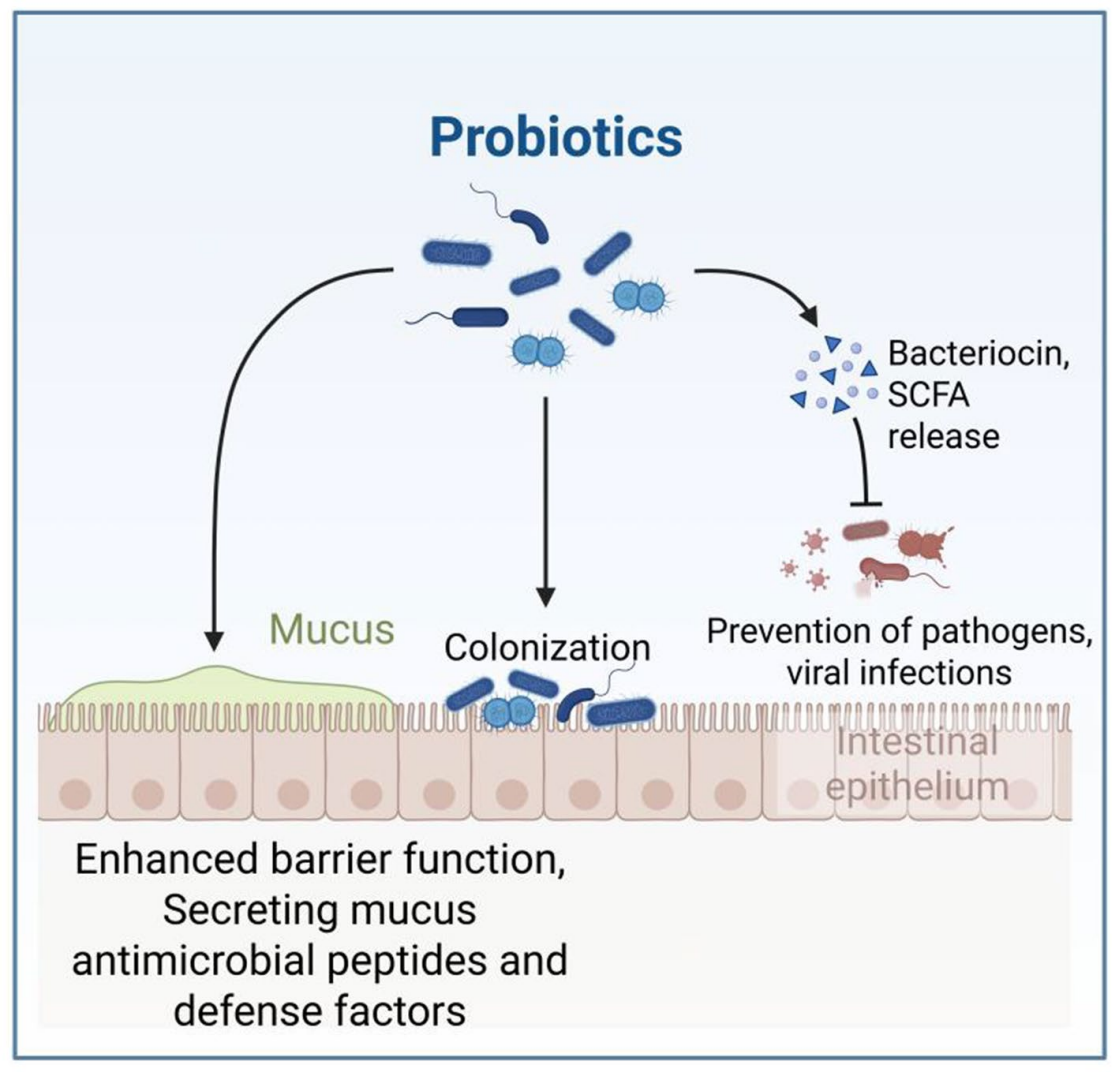
This figure demonstrates how probiotics function in the intestinal environment, showing their dual mechanisms of action: direct colonization of the intestinal epithelium and enhancement of the mucosal barrier through bacteriocin/SCFA production that prevents pathogen invasion. The figure is created with BioRender.com.

**Figure 7:**
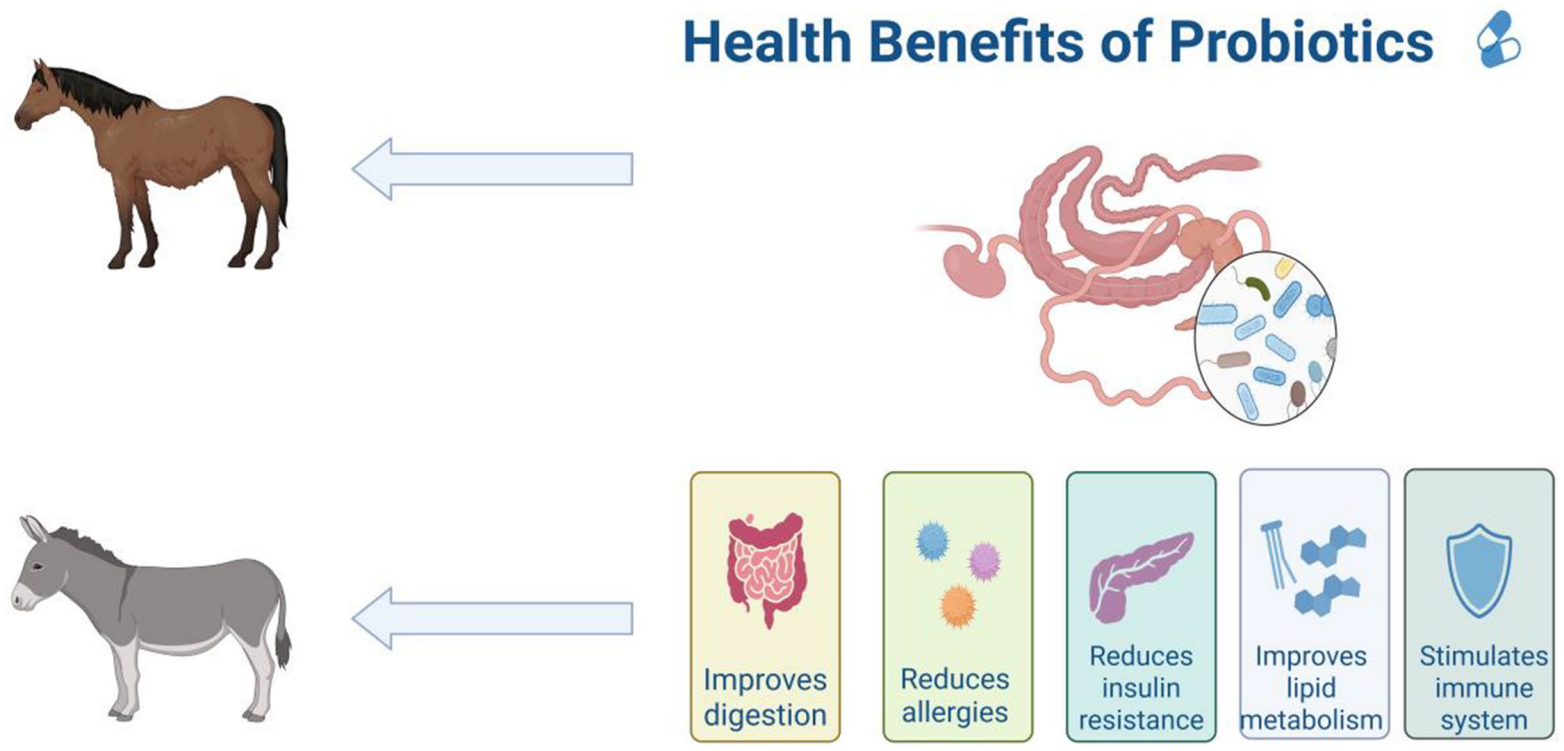
Benefits of probiotics in the gut for equine animals, including improved digestion, reduced allergies, reduced insulin resistance, improved lipid metabolism and stimulated immune system. The figure is created with BioRender.com.

Probiotics have been widely studied for their humoral, cellular, and non-specific immune regulatory effects (Gill et al., 2001). Probiotics attach to the animal intestine to form a mucosal barrier, secreting antibiotics and other substances to prevent pathogen and viral infections; Stimulate and regulate various intestinal functions, including digestion, metabolism, immune response, and pathogen elimination, and affect the brain gut axis (Figure 6; Conca, 2016; Kristensen et al., 2016). Probiotics can also release cytokines such as interleukin (IL), tumor necrosis factor (TNF), interferon (IFN), and transforming growth factor (TGF) to regulate innate and adaptive immune responses (Figure 7; Foligné et al., 2010). The non-toxic metabolites produced by the gut microbiota play important roles in both nutritional and clinical environments (Kau et al., 2011; Bermudez-Brito et al., 2012; Bin et al., 2018). By fermenting food that is not easy to digest, probiotics provide energy and show antigenicity, anti-obesity, anti-diabetes and anti-inflammatory properties (Kerry et al., 2018). These findings emphasize the crucial role of gut microbiota metabolites in regulating host immune function and gut immune response. Given the complexity of microbial interactions in mediating these processes, further research on how gut microbiota metabolites affect immune system regulation remains crucial.

The gut microbiota includes multiple types, and different bacterial strains have unique effects on immune regulation. Beneficial bacteria such as *lactobacilli* and *bifidobacteria* can promote intestinal health; Harmful bacteria such as *Escherichia coli* and *Staphylococcus aureus* can damage the intestinal environment; Neutral bacteria such as *Firmicutes* and *Bacteroidetes* are harmless under normal circumstances, but may cause diseases under specific conditions. These microorganisms are crucial for maintaining the intestinal immune barrier. Some typical bacterial strains have been confirmed to have immune regulatory functions. Research has found that *Bifidobacterium* binds tightly to mammalian intestinal mucosal epithelial cells through phosphorylated soap acid, forming a biological barrier on the intestinal mucosal surface together with other anaerobic bacteria, effectively resisting various pathogenic bacteria and harmful microorganisms (Alessandri et al., 2021). This indicates that gut microbiota has multiple effects on the host immune system, but existing research has only revealed a small part of the mechanisms and has not yet formed a complete system. More research is needed in the future to verify the specific roles of different gut microbiota in the immune system of horses.

## 5 Regulation of gut microbiota in equine animals

### 5.1 Environmental factors

The gut microbiota is the “extra genome” of horses, which is related to their health through multiple pathways. Under normal circumstances, the composition of the gut microbiota in horses is influenced by various environmental factors, including management factors, seasonal changes, and social interactions, in a symbiotic and dynamic relationship with the host. When designing and interpreting research on the gut microbiota of horses, these factors must be considered. Research has shown that the composition of microbial communities presents a multi-level structure, with significant differences observed at the individual, population, and spatial levels (Antwis et al., 2018). The microbiota of horse feces and skin tends to vary depending on geographical location and habitat. Consistently, Ayoub et al. (2022) found significant differences in protein content in the fecal microbiota of healthy horses (teaching horses vs. client owned) raised in different facilities (Ayoub et al., 2022), demonstrating the impact of management and environment on their fecal microbiota Finally, the higher similarity in the skin microbiota of horses living on the same farm reinforces the conclusion that the environment is an important contributor to the seeding of bacterial species in horses (Kaiser-Thom et al., 2021). It has been reported in study by Salem et al. (2018) that seasonal changes in the fecal microbiota of horses raised on pastures for 12 months, with management measures remaining largely unchanged. Their findings indicate that seasonal changes, weather conditions (temperature and rainfall), and additional feed intake (age) are associated with microbial fluctuations. These results highlight the dynamic characteristics of the gut microbiome in horses and their ability to respond to environmental changes. To evaluate whether these seasonal effects are consistent over time, further longitudinal studies lasting at least 24 to 36 months are needed.

A study by Salem et al. (2018) demonstrated that the gut microbiota of equines varies throughout the year. Although feed and variety significantly affect gut microbiota, some microbial food sharing and social interactions may partially explain these changes (Garber et al., 2020). A study investigating semi local Welsh contributions found that although individual variation accounted for 52.6% of gut microbiota variation, group interaction and social behavior also contributed to overall variation (Antwis et al., 2018). Research has found that mother son relationships have a significant impact on microbial community similarity, with foals having a higher similarity to their mother’s microbiota than to other mare populations, indicating to some extent that their microbiome composition may be influenced by each other (Boucher et al., 2024).

### 5.2 Dietary factors

#### 5.2.1 High fiber and starch dietary components

Dietary composition serves as a fundamental regulatory mechanism for equine gut microbiota modulation (Zhang et al., 2022e). Specifically, diets characterized by elevated animal protein and fat content have been demonstrated to promote increased abundance of *Firmicutes* within the rectal microbiota. Conversely, high-fiber dietary regimens facilitate enhanced concentrations of *Bacteroidetes* and fiber-degrading bacterial populations (De Filippo et al., 2010; Wu et al., 2011). Cultivation-based microbial investigations have further elucidated the metabolic shifts occurring during dietary transitions. When equines transition from high-fiber to high-starch diets, a notable increase in the abundance of starch-degrading bacteria, lactic acid-metabolizing bacteria, and Streptococcus species occurs within the gastrointestinal tract. Simultaneously, this dietary shift results in a corresponding decrease in fiber-degrading bacterial populations (Raspa et al., 2024). This microbial transformation can be attributed to the physiological limitations of starch digestion in equines. When horses consume excessive starch quantities (exceeding 1 gram per kilogram of body weight per meal), substantial amounts remain undigested within the foregut (Harris and Dunnett, 2018). Subsequently, this undigested starch undergoes fermentation within the hindgut (Richards et al., 2006), resulting in elevated lactate production. This metabolic environment preferentially supports bacterial communities specialized in starch-to-lactate conversion, while simultaneously creating conditions unfavorable for beneficial fiber-fermenting microorganisms, thereby reducing fiber-degrading bacterial populations. In contrast, high-fiber dietary regimens demonstrate the capacity to enhance fiber-degrading bacterial abundance within equine gut microbiota (Grimm et al., 2017). Comparative studies examining the effects of high-fiber versus high-starch diets on apparent digestibility in donkeys revealed superior digestibility coefficients for high-fiber dietary interventions. Additionally, yeast polysaccharides (YPS) supplementation has been shown to exert beneficial effects on the fecal microbiome, specifically promoting the proliferation of advantageous microorganisms including *Lactobacilli* and *Prevotella* species in donkey foals (Huang et al., 2023). Recent metagenomic analyses conducted by Cai et al. (2024) investigated the influence of concentrate feeding sequences on gut microbiota diversity in fattening donkeys. Their findings demonstrated that initial concentrate feeding resulted in increased abundance of beneficial bacterial phyla, particularly *Bacteroidetes* and *Firmicutes*. These microbial communities play pivotal roles in carbohydrate metabolism and nutrient processing, thereby contributing to the maintenance of optimal digestive function. Furthermore, Zhang et al. (2021) reported that high-energy dietary interventions significantly reduced the *Firmicutes* to *Bacteroidetes* (F/B) ratio. Additionally, high-energy diet administration resulted in decreased alpha diversity within the rectal microbiota, reduced relative abundance of unidentified *Ruminococcaceae* species, and increased abundance of specific microorganisms within the Actinobacteria, *Rikenellaceae,* and *Veillonellaceae* genera.

#### 5.2.2 Methionine supplementation effects on gut microbiota

Methionine supplementation has been demonstrated to enhance lactation performance in donkeys through complex microbiota-mediated mechanisms. Huang et al. (2024) established that dietary methionine addition improves lactation capacity by increasing both milk production and component synthesis. This enhancement occurs through the regulation of serum metabolites, which subsequently improves milk quality and donkey milk metabolite profiles, ultimately optimizing lactation performance. The underlying mechanism involves gut microbiota compositional modifications that influence mammalian milk production and composition. Subsequent research by Huang et al. (2025) revealed that methionine supplementation in lactating donkeys significantly increases the abundance of *Methanococcus, Ruminococcaceae*, and fiber-degrading bacteria, while simultaneously reducing *Ruminococcaceae* populations. Notably, methanogenic bacteria demonstrate associations with organismal antioxidant enzyme systems (Wu et al., 2021). In addition, methionine supplementation significantly enhanced serum antioxidant activity in lactating donkeys, with antioxidant indices (T-AOC and CAT activity) showing correlations with microbial abundance patterns (Guo et al., 2024). Specifically, *Ruminococcus* species exhibited significant positive correlations with T-AOC and CAT activity, while methanogenic bacteria demonstrated positive correlations with T-AOC levels.

#### 5.2.3 Weaning-associated microbial changes

Weaning represents a critical developmental phase in animal production, characterized by substantial microbial compositional changes within the digestive tract in response to multiple biological and environmental stressors (Zhang et al., 2024). Prior to weaning, *Methanobacterium* constitutes the predominant archaeal genus in both donkey and foal fecal samples, indicating that initial colonization and establishment of methanogenic bacterial populations commence before the weaning process (Zhang et al., 2023). Microbial analysis of donkey and foal fecal samples reveals characteristic diversity patterns during the weaning transition (Yang et al., 2024). Microbial diversity typically exhibits an initial decrease followed by subsequent recovery, with significant reductions observed before and during weaning. This pattern likely reflects effective microbial adjustment and adaptation to environmental changes as foals transition from liquid milk to combined liquid and solid feed consumption. The significant increasing trend observed from weaning through post-weaning periods may be attributed to foal adaptation to solid feed consumption. However, the gut microbiota represents an inherently fragile ecosystem, and the extensive dietary modifications accompanying weaning can precipitate dramatic microbial community alterations. These microbial shifts often result in increased abundance of diarrhea-associated bacteria within the equine intestinal tract, potentially triggering stress-induced diarrheal responses.

Recent investigations by Xie et al. (2023) employed high-throughput 16S rRNA sequencing technology to examine the effects of concentrate feeding sequences on intestinal microbiota richness in weaned donkeys (6-month-old males). Their results demonstrated that the concentrate-crude mixed diet (C3) treatment produced optimal growth performance in weaned donkeys, accompanied by increased relative abundance of *Bacteroidetes* and *Spirochaete*s. Given the high cellulose-degrading capacity of *Spirochaetes*, these findings suggest that concentrate-crude mixed diets promote fibrous substrate degradation by intestinal microbiota through enhanced *Spirochaetes* populations, thereby facilitating optimal nutrient absorption and growth rates in donkeys.

### 5.3 Genetic and variety factors

Research indicates both similarities and differences in the composition of the gut microbiota across various horse breeds. Comparative studies of Mongolian and Thoroughbred horses have identified notable patterns in microbial communities. The predominant bacterial phyla in both breeds are *Firmicutes* (56% in Mongolian horses, 53% in Thoroughbred horses) and *Bacteroidetes* (33% in Mongolian horses, 32% in Thoroughbred horses), with these taxa constituting the major components of the gut microbiota in both breeds. Despite these similarities, some key differences in bacterial composition were observed. Specifically, Eutrophic bacteria, *Planctomycetes* (floating molds), *Proteobacteria*, *TM7*, and *Chloroflexi* were significantly more abundant in Thoroughbred horses compared to Mongolian horses (Zhao et al., 2016). Conversely, the *Treponema* genus was more abundant in Mongolian horses (43%) compared to Thoroughbreds (29%) (Guo et al., 2022).

Breed-specific differences in the gut microbiota have been linked to distinct clinical conditions. For instance, Arabian horses are predisposed to colic (Gonçalves et al., 2002), while dwarf horses exhibit a higher susceptibility to glucose intolerance, obesity, and metabolic syndrome (Johnson, 2002). These variations in gut microbiota between breeds may influence disease susceptibility and could contribute to the occurrence of stereotypical behaviors in certain equine populations.

A study used high-throughput sequencing to compare the richness of gut microbiota in the 16rDNA V3-V4 variable region between wild and domesticated donkeys (Costa and Weese, 2018). The results indicate that the microbial community structure of wild donkeys is more complex than that of domesticated donkeys, and their dry matter digestibility is also higher. In addition, they found that the composition and functional characteristics of this microbial community may be closely related to its long-term adaptation to the extreme high-altitude environment of the Tibet Plateau under selection pressure, and can be inherited by offspring (Costa et al., 2012). Liu et al. (2020) found that the two dominant phyla of wild donkeys, *Bacteroidetes* and *Firmicutes*, were significantly higher than those of domestic donkeys, while wild donkeys had *Ruminococcocacae NK4A214*, *Phascolarctobacteria*, *Coprostanogenes*, *Lachnospiraceae XPB1014 Akkermansia* is significantly higher than domestic donkeys, highlighting the key role of specific species differences in the gut microbiota of animal physiology and health.

Further studies on different equine animals such as donkeys and horses have shown significant differences in the microbial composition of their gut microbiota. Liu (2020) reported that the ratio of *Firmicutes* to *Bacteroidetes* in donkeys is approximately 1:1, while in horses it is 0.8:1. The ratio of *Firmicutes/Bacteroidetes* has been shown to affect obesity in animals, with higher ratios promoting increased energy absorption and fat storage (Bell, 2015; Ley et al., 2003). Liu (2020) confirmed these findings, demonstrating that under the same feeding conditions, donkeys tend to accumulate more fat than horses. This difference may be due to the specific metabolic functions of these bacterial phyla: *Bacteroidetes* mainly metabolize steroids, bile acids, and polysaccharides, while *Firmicutes* focuses on fermenting carbohydrates. Interestingly, animal methane emissions may be partially influenced by diet, but are also related to genetic factors. Methanogenic bacteria are widely regarded as the most heritable gut microbiota in humans and various animal populations (Grieneisen et al., 2021), and are also representative archaea in the cecum of horses. In the intestine of horses, the fermentation site is mainly in the cecum (pH 6–7), with bacteria dominating (mainly *Firmicutes* and *Bacteroidetes*), a low proportion of archaea, and weak methane production pathways. The dominant metabolites are acetic acid and propionic acid, and the peak gas production during fermentation is early with weak persistence. In contrast, the intestine of donkeys is mainly fermented in the cecum and colon (Zhang et al., 2022d), with a large number of anaerobic fungi (such as *Ruminococcus*). The diversity of archaea is low, and the gas production during fermentation is gentle and long-lasting. It can effectively degrade cellulose, prolong the retention time of chyme, and improve fiber digestion rate. For ease of reference, Table 2 shows the differences in gut fermentation patterns and microbiota between horses and donkeys (supplemented with the fermentation patterns of ruminants as a reference). These data are sourced from previously published articles (Liu, 2020; Li et al., 2023; Beckers et al., 2017; McCann et al., 2017). Stothart et al. (2024) found in their study of Sable Island horses that microbiome characteristics closely related to survival have higher interannual reproducibility among individuals. This result may indicate that, like other mammals, microorganisms associated with intestinal methane production may have a partially accumulated genetic basis or be influenced by the preferential effect of Sebur Island horses.

**Table 2:** Differences in gut fermentation patterns and microbiota between horses and donkeys.

Feature	Horse	Donkey	Ruminant
Fermentation site	Posterior intestine (mainly cecum)	Posterior intestine (cecum+colon)	Rumen (front-end fermentation)
Advantageous metabolites	Acetic acid, propionic acid	Acetic acid and butyric acid (higher in low-quality feed)	Acetic acid, propionic acid, methane
Microbial diversity	Bacteria dominate, with a low proportion of archaea	Anaerobic fungi are abundant, while archaeal diversity is low	High proportion of archaea (methanogens)
PH adaptability	Cecum pH 6–7 (weakly acidic)	Similar to horses, but with stronger acid resistance	Rumen pH 5.5–6.5 (acidic)
Fermentation persistence	Early peak gas production with weak sustainability	Smooth gas production with a long duration	Continuous high gas production (dependent on fiber degradation)

Additionally, it is important to consider the impact of factors such as the health status and management of equine animals on their gut microbiota composition and structure. The gut microbiota of different breeds of animals can also vary greatly in an unhealthy state, so individual and breed differences, as well as differences between age groups, can also make the process of defining the microbiota of “healthy” or “diseased” equines complex (Murcia, 2019). It is noteworthy that metagenomic studies alone cannot provide a complete understanding of the functional pathways of microbial communities (Marchesi and Ravel, 2015). Without complementary metabolomic analyses, it is difficult to establish causal relationships between changes in microbial composition and physiological outcomes in horses. Metabolomics, which provides direct insights into cellular activity by profiling metabolites in a given environment, holds significant potential for elucidating the functional significance of microbial communities and their impact on host physiology (Garber et al., 2020; Li et al., 2023). For ease of reference, Table 3 and Figure 1 show various comparisons of regulatory factors of the gut microbiota in equine animals, and list the references.

**Table 3:** Factors affecting gut microbiota in equine animals.

Factors	Comparison of different factors in the same aspect	Effect on gut microbiota	Species	Reference
Environmental factors	Management factors	Protein content in fecal microbiota varies among horses raised in different facilities Hay feeding increases the relative abundance of *filamentous bacteria* and *spirulina* Wild donkeys are superior to domestic donkeys in terms of dry matter digestion, gut microbiota composition, and function	Horse and Donkey	Ayoub et al. (2022), Kaiser-Thom et al. (2021), and Khan et al. (2024)
	Seasonal changes	Weather conditions influence both feed availability and quality for horses, as well as environmental microbial populations. Ingested environmental bacteria can colonize the hindgut, leading to changes in the gut microbiota.	Horse	Salem et al. (2018)
	Social interactions	Individual variation accounts for 52.6% of the variation in the gut microbiota Group interaction leads to overall variation in gut microbiota Social behavior leads to overall variation in gut microbiota	Horse	Antwis et al. (2018)
Dietary factors	High fiber and high starch	A high starch diet has a high abundance of starch degrading bacteria, lactic acid degrading bacteria, and streptococcus A high fiber diet with a large number of fiber degrading bacteria A high fiber diet has higher apparent digestibility than a high starch diet A high energy feed significantly reduces the ratio of *Firmicutes* to *Bacteroidetes* (F/B)	Horse	De Filippo et al. (2010), Wu et al. (2011), Raspa et al. (2024), and Zhang et al. (2021)
	Methionine in the diet	High Met increases the abundance of ruminant bacteria, methane bacteria, etc. High Met can enhance the antioxidant capacity of lactating donkeys	Donkey	Huang et al. (2024) and Huang et al. (2025)
	Weaning	Diversity of microorganisms in weaned donkeys first decreased and then increased Weaned donkeys and foals fed a mixed diet of fine and coarse grains, with high relative abundance of *Bacteroidetes* and *Spirobacteria*	Horse Donkey	Yang et al. (2024) and Xie et al. (2023)
Genetic and variety factors	Different horse varieties	*Synergistetes*, *Planctomycetes*, *Proteobacteria*, *TM7*, and *Chloroflexi* were significantly more abundant in Thoroughbred horses Treponema genus was more abundant in Mongolian horses (43%). Arabian horses are predisposed to colic Dwarf horses exhibit a higher susceptibility to glucose intolerance, obesity, and metabolic syndrome	Horse	Gonçalves et al. (2002) and Johnson (2002)
	Different donkey varieties	Wild donkeys have a high digestibility of dry matter. Dry matter digestion rate of domestic donkeys is low Wild donkeys also exhibit more complex microbial communities. The *Bacteroidetes* and *Firmicutes* phyla of wild donkeys are higher than those of domestic donkeys The *Ruminococcaceae NK4A214*, *Phascolarctobacteria*, *Coprostanogenes*, *Lachnospiraceae XPB1014*, and *Akkermansia* in wild donkeys are significantly higher than those in domestic donkeys	Donkey	Costa and Weese (2018) and Liu et al. (2020)
	Different equid species	The ratio of *Firmicutes* to *Bacteroidetes* in donkeys is approximately 1:1, compared to a ratio of 0.8:1 in horses. Specific metabolic function of the bacterial phylum causes donkeys to accumulate more fat than horses In the intestines of horses, the proportion of archaea is low and the methane production pathway is weak, while donkeys have a large number of anaerobic fungi in their intestines	Horse and Donkey	Liu (2020), Liu et al. (2020), and Li et al. (2023)

Recent studies have significantly advanced our understanding of the complex and diverse microbial communities in the equine intestinal tract, which not only enhances our comprehension of the role of gut microbiota in animal health but also provides a theoretical framework for future interventions. In equines, the core microbial flora of the intestinal tract is essential for the digestion and breakdown of feed nutrients, immune regulation, disease prevention, and overall health maintenance (Guo et al., 2022). These microbial communities play a crucial role in cellulose degradation and the production of short-chain fatty acids, which are vital for nutrient absorption and energy metabolism in equine species (Liu, 2020). Furthermore, the core microbiota contributes to host resistance against pathogen invasion, supports immune system function, and maintains intestinal health by regulating immune responses and preserving the integrity of the intestinal barrier.

Notably, the composition of the intestinal microbiota has been closely associated with the health status of equines. Future research should focus on the influence of environmental factors—such as geographical location, seasonal variations, and dietary composition—on the core microbial flora of equines. This could offer insights into how to optimize equine health and performance. Additionally, understanding the diversity and richness of the gut microbiota, as well as its impact on nutrient absorption, will be instrumental in improving the management practices of equine animals.

## 6 Differences in digestive capacity between horses, ruminators, and humans

When comparing the intestinal microbiota of equines with other species, such as ruminants and humans, notable differences arise. While equines, like ruminants (e.g., cattle, sheep), and humans possess complex microbial communities, the specific functions and mechanisms of action vary. The intestinal system of equines differs from that of ruminants, particularly in the absence of a rumen, reticulum, omasum, and abomasum. These specialized compartments in ruminants facilitate the efficient digestion of cellulose-rich plant materials, supported by abundant microbial populations that break down indigestible plant fibers and produce volatile fatty acids as a primary energy source (Campos et al., 2016). In contrast, equines have a relatively small stomach but a relatively long small and large intestine, particularly the highly developed cecum, which compensates for the lack of a rumen by facilitating cellulose digestion. However, this digestive process is less efficient than in ruminants due to the absence of rumination, which allows for additional chewing and reprocessing of food to enhance microbial action (David et al., 2014).

Equine species primarily rely on microbial fermentation to digest plant fibers, while humans utilize digestive enzymes for food breakdown. This structural and functional divergence reflects an adaptive specialization of equines for plant fiber digestion, although this may limit their efficiency in digesting other food types. The human digestive system, by contrast, is relatively short and adaptable, optimized for a varied diet. While equine digestive systems are designed for fibrous plant material, they are less flexible for processing other food types, highlighting the concept of structural adaptation to dietary function.

At present, there is a lack of detailed comparative data on the intestinal microbiota of equine animals versus other species. Future studies could provide valuable insights into the similarities and differences between the gut microbiota of equines and other animals, contributing to the enhancement of equine health, productivity, and evolutionary understanding. Such research could also deepen our knowledge of intestinal microbiomes in other species, facilitating a broader understanding of host–microbe interactions.

## 7 Future perspectives

Looking ahead, with ongoing advances in research and technological innovation, we anticipate gaining deeper insights into the core gut microbiota of equine animals. This could offer novel strategies for improving animal health, feed efficiency, and exercise performance. The adaptive coevolution of intestinal microbes with their hosts suggests that gut microbiota plays a significant role in host metabolism, immunity, and disease prevention. For example, intestinal microbes are involved in the digestion of plant polysaccharides and the detoxification of food-derived toxins (Kikuchi et al., 2012; Yoo et al., 2020). They also influence host metabolism, immune responses, and cardiovascular health, with emerging evidence showing that dysbiosis of the gut microbiota may contribute to conditions like hypertension (Li et al., 2017; Wilck et al., 2017). During animal evolution, the gut microbiota has played an integral role in the development of host species (Belkaid and Hand, 2014). Therefore, intestinal microbes should be viewed not only as symbiotic organisms but as essential components of animal physiology and behavior. Future research could explore the dynamics of gut microbial communities in equines, particularly their relationships with host immune systems, and how they contribute to maintaining intestinal homeostasis.

To advance our understanding, future studies could investigate the diversity and functional roles of microbial communities across different equine species, management practices, and environmental conditions. Metagenomic approaches can be employed to study the intestinal microbiota in more detail, helping to elucidate the specific contributions of microorganisms to host health. Additionally, regulating the gut microbiota through feed additives or probiotics could be explored as a potential means of enhancing equine health, performance, and feed efficiency. Understanding how microbial communities respond to seasonal and dietary changes and their associated physiological effects is another critical area for investigation.

Therefore, in the future, research on the gut microbiota of equine animals can make breakthrough progress through multi omics techniques. By integrating spatial transcriptomics and single-cell metabolomics, a spatial localization map of microbial host cell interactions can be drawn to reveal the molecular mechanism of key metabolites (such as butyrate) activating the intestinal epithelial energy pathway through G protein coupled receptors. At the same time, we need to break through the bottleneck of anaerobic bacteria *in vitro* culture technology and establish a single-cell database of equine animals; Based on cross species comparative genomics, screening for host genetic microbial co evolutionary markers (such as the synergistic relationship between *KEAP1* gene mutations and abundance of Mongolian horse butyric acid producing bacteria) provides a new target for stress resistant breeding.

At the application level of transformation, the focus will be on precise regulation of athletic performance: based on the enrichment of butyric acid producing bacteria in horse racing, engineering microbial preparations against gastric acid embedding will be developed to promote the biosynthesis of muscle mitochondria through the “gut muscle axis” mechanism; For metabolic diseases, we will analyze the interference mechanism of the microbiota bile acid axis on the FXR/FGF19 pathway under high-fat diet, and use bacteriophages to selectively clear pathogenic streptococcus to inhibit inflammation; We aim to enhance the stress resistance of domestic donkeys through microbiota transplantation by utilizing the characteristic of Akman bacteria to enhance the intestinal barrier of wild donkeys.

Specific intervention measures for equine animals require multidimensional strategies, such as developing tannin cellulose composite slow-release feed to balance the microbial community, adding seasonally adapted microbial agents (such as succinic acid metabolizing bacteria) to stabilize intestinal pH, constructing a comprehensive prevention and control system for antibiotic resistance genes, replacing antibiotics with antimicrobial peptides, targeting CRISPR-Cas9 nanoparticles to eliminate antibiotic resistance plasmids, and introducing anaerobic bacterial niches to inhibit the spread of antibiotic resistant bacteria; Optimize microbiome transplantation technology, establish screening criteria for “super donors,” and develop oxygen tolerant bacterial powder capsules.

Applying synthetic biology to construct engineering yeast for large-scale production of short chain fatty acids, while establishing a “microbial diversity red line” for wild horse species to maintain ecological adaptability, ultimately forming a three-dimensional mechanism analysis precise intervention ecological maintenance model: the basic layer draws host microbial holograms, the application layer develops targeted delivery systems and gene editing tools to prevent metabolic diseases, and the ecological layer establishes a germplasm resource microbial library to support genetic diversity protection, thereby connecting the causal chain of “microbial gene host phenotype” and promoting the transformation of the horse industry towards data-driven management.

## 8 Conclusion

In conclusion, the investigation of the core gut microbiota in equine animals holds significant scientific and practical value. A deeper understanding of the microbial composition and functional dynamics of the equine intestinal tract offers potential for the regulation and optimization of gut health. By refining environmental conditions and dietary strategies, it may be possible to prevent or treat gut-related disorders effectively. This approach promises to enhance the health management and sustainable development of the equine industry. As research advances and technologies progress, the ability to harness and manipulate intestinal microbial resources will likely play a pivotal role in furthering the welfare and productivity of equine populations, contributing to the long-term success of the industry.
